# Manual therapy interventions in the management of adults with prior cervical spine surgery for degenerative conditions: a scoping review

**DOI:** 10.1186/s12998-022-00422-8

**Published:** 2022-03-07

**Authors:** Jordan A. Gliedt, Aprill Z. Dawson, Clinton J. Daniels, Antoinette L. Spector, Zachary A. Cupler, Jeff King, Leonard E. Egede

**Affiliations:** 1 Center for Advancing Population Science, Medical College of Wisconsin, WI Milwaukee, USA; 2 Department of Neurosurgery, Medical College of Wisconsin, Milwaukee, WI USA; 3 Division of General Internal Medicine, Department of Medicine, Medical College of Wisconsin, Milwaukee, WI USA; 4 VA Puget Sound Health Care System, Tacoma, WA USA; 5 Institute for Health and Equity, Medical College of Wisconsin, Milwaukee, WI USA; 6 Butler VA Health Care System, Butler, PA USA; 7 Institute for Clinical Research Education, University of Pittsburgh School of Medicine, Pittsburgh, PA USA

**Keywords:** Postsurgical, Postoperative periods, Cervical post-surgical syndrome, Spinal manipulation, Manual therapy

## Abstract

**Objective:**

Cervical spine surgeries for degenerative conditions are rapidly increasing. Cervical post-surgery syndrome consisting of chronic pain, adjacent segment disease, recurrent disc herniation, facet joint pain, and/or epidural scarring is common. Repeat surgery is regularly recommended, though patients are often unable to undergo or decline further surgery. Manual therapy is included in clinical practice guidelines for neck pain and related disorders, however clinical guidance for utilization of manual therapy in adults with prior cervical spine surgery is lacking. This study aimed to synthesize available literature and characterize outcomes and adverse events for manual therapy interventions in adults with prior cervical spine surgery due to degenerative conditions.

**Methods:**

Preferred reporting items for systematic reviews and meta-analyses extension for scoping reviews was followed. PubMed, Cumulative Index of Nursing and Allied Health Literature, physiotherapy evidence database, and Index to Chiropractic Literature were searched from inception through October 2021. English-language literature comprised of randomized clinical trials (RCT), case–control, cohort, and case report designs were included. Adults undergoing manual therapy, with or without combination of other interventions, with prior cervical spine surgery due to degenerative conditions were included.

**Results:**

Twelve articles were identified, including 10 case reports, 1 low-quality RCT, and 1 acceptable-quality RCT. Eight case reports described 9 patients with history of fusion surgery. Two case reports described 2 patients with history of discectomy. One case report described one patient with separate operations of a discectomy at one level and a fusion at another level. One case report described 2 patients with history of cervical disc replacement surgery. The two RCTs included 63 and 86 participants, respectively. Use of manual joint mobilization/manipulation, table/instrument assisted mobilization/manipulation, and multimodal interventions were described in eligible studies. Favorable clinical outcomes were reported in 10 studies. Six case reports/series involving 8 patients described use of unclassified forms of manual therapy. Eight studies described the use of multimodal interventions along with manual therapy. One study described high patient satisfaction. Two studies, accounting for 3 patients, reported serious adverse events.

**Conclusions:**

There is a lack of literature informing evidence related to clinical outcomes, patient satisfaction, and adverse events associated with manual therapy for patients with prior cervical spine surgery due to degenerative conditions. High-quality studies of higher-level hierarchical study design are needed to understand the clinical utility and safety profile of manual therapy for this population.

**Supplementary Information:**

The online version contains supplementary material available at 10.1186/s12998-022-00422-8.

## Introduction

Cervical spine surgery is a common and increasingly performed intervention for degenerative conditions of the cervical spine [1–8]. Surgical intervention for cervical degenerative conditions is one of the leading elective surgical procedures performed in the United States [1, 3]. Rates of cervical fusion surgeries have seen a particularly significant increase [7, 8], with anterior cervical discectomy and fusion (ACDF) reported as the most commonly performed surgical procedure for degenerative cervical spine conditions [1, 9]. Total cases of cervical fusion and cervical decompression surgeries for cervical degenerative pathology has been measured at 60.8 cases per 100,000 adults in the United States [3].

Studies have suggested between 13 and 32 percent of cervical spine surgeries result in difficulties, such as cervical post-surgery syndrome and require repeat surgery [10, 11], including a potential incidence of 2.9 percent per year requiring repeat cervical spine surgery due to symptomatic adjacent segment disease [12, 13]. Subsequently, a subset of individuals may experience ongoing symptoms associated with cervical post-surgery syndrome, which may include chronic axial pain with or without radicular symptoms, adjacent segment disease, recurrent disc herniation, facet joint pain, and epidural scarring [13]. Despite the potential need for repeat cervical spine surgery, there is patient and clinician variability in decision making related to when to proceed with repeat cervical spine surgery [11, 14].

Manual therapy is a non-operative intervention aimed at assessing, diagnosing, and treating a variety of musculoskeletal and spine related complaints [15]. Multiple types of techniques constitute manual therapy, though it is generally categorized into four main groups: (1) joint mobilization, (2) joint manipulation, (3) static or passive musculoskeletal stretching, and (4) manual or instrument assisted soft tissue manipulation [15]. Evidence is emerging as favorable for the use of manual therapy in cervical related conditions, including chronic neck pain [15, 16], with manual therapy recommended in clinical practice guidelines as a management strategy for individuals with these conditions [17, 18].

It is conceivable that manual therapy might be an effective management option for individuals with prior cervical spine surgery for degenerative conditions, though there is a paucity of literature available to guide clinical decision making on utilization of postoperative manual therapy. We are unaware of any prior literature synthesizing the evidence on outcomes or safety profile for manual therapy in individuals with prior cervical spine surgery. Therefore, the purpose of this study was to synthesize the literature regarding types of manual therapy employed, and outcomes and adverse events for manual therapy interventions in adults with prior cervical spine surgery due to degenerative conditions.

## Methods

Consistent with recommendations by Munn et al. [19] a scoping review approach was selected with an aim to assess the state of the current literature, identify knowledge gaps, and analyze characteristics related to an individual concept—outcomes and safety profile associated with varying types of manual therapy interventions for individuals with prior cervical spine surgery due to degenerative conditions. The Preferred Reporting Items for Systematic Reviews and Meta-Analyses extension for Scoping Reviews (PRISMA-ScR) checklist was followed [20]. The PRISMA-ScR checklist is included as a supplement to this manuscript. This scoping review was conducted in 5-stages and in accordance with methodology described by Arskey and O’Malley [21] and later revised by Levac [22]. This review did not conduct a sixth stage—consultation—as this stage is considered optional [21]. This review was not registered prior to undertaking it as protocols do not require registration of scoping reviews.

### Stage 1: Identifying the research question

This review addressed the following research question: *What are the outcomes (e.g. pain, function, disability, medication consumption, patient satisfaction) and adverse events associated with manual therapy interventions for adults with prior cervical spine surgery due to degenerative conditions?*

### Stage 2: Identifying relevant studies

**Fig. 1:**
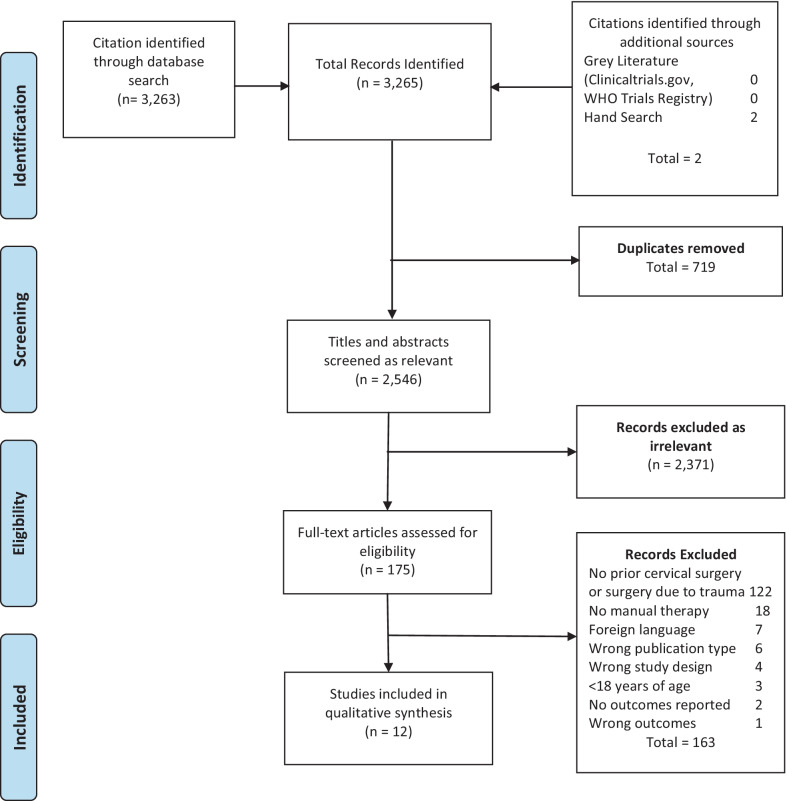
PRISMA flow diagram

A literature search was performed on May 2, 2020 and updated on October 21, 2021 of the following databases from inception through October 21, 2021: PubMed, Cumulative Index of Nursing and Allied Health Literature (CINAHL), Physiotherapy Evidence Database (PEDro), and Index to Chiropractic Literature (Fig. 1). Author expertise, the Cochrane Back and Neck Group guideline for systematic reviews [23] and prior related Cochrane reviews [24–26] were used to direct our search strategy. A variety of search terms related to manual therapy intervention, surgical intervention, and health condition/body region were combined for the database search (Table 1). Investigators were asked to identify additional studies in which they were familiar, but which were missing from the formal search. There was an attempt to identify completed studies accepted for publication though not yet in print via search of clinicaltrials.gov and the World Health Organization (WHO) International Clinical Trials Registry. A hand search was performed to identify additional articles not identified through the database search. Literature identified in this search was downloaded to EndNote X9 for Windows and duplicates were removed.

**Table 1:** Search strategy and search example of the PubMed database

Treatment strategy	Prior procedure	Condition/region
-Chiropractic -Chiropractor -Chiropractic adjustment -Musculoskeletal Manipulations -Osteopathic manipulations -Orthopedic manipulations -Manual therapy -Manual therapies -Manipulative therapy -Manipulative therapies -Manipulative rehabilitation -Joint manipulation -Joint mobilization -Mobilization therapy -Spinal mobilization -Spinal manipulative therapy -Cervical manipulation -Cervical mobilization -Soft tissue mobilization -Flexion-distraction -Myofascial -Active release -Graston -Massage -Stretching techniques -Muscle stretching -Static stretching -Passive stretching -Proprioceptive Neuromuscular facilitation -PNF stretching -Post isometric relaxation -Contract-relax -Instrument assisted soft tissue -Instrument assisted manipulation -Instrument assisted adjustment -Instrument assisted adjusting -Manipulation under anesthesia -Spinal manipulation -Muscle energy technique	-Arthrodesis -Postsurgical -Postoperative -Post-surgical -Post-operative -Fusion -Spinal fusion -Cervical fusion -Decompression -Cervical spine surgery -Microdiskectomy -Microdiscectomy -Discectomy -Diskectomy -Laminectomy -Laminotomy -Osteotomy -Disc replacement -Disk replacement -Artificial disc replacement -Vertebroplasty -Kyphoplasty -Foraminotomy -Interlaminar implant -Spinal cord stimulator -Intrathecal drug delivery -Laser surgery -Interbody -Minimally invasive spine Surgery -Surgery -Surgical	-Failed back syndrome -Cervical post surgery syndrome -Post surgery syndrome -Spine -Spinal-cervical vertebrae -Cervical -Cervicalgia -Cervical pain -Degenerative -Degeneration -Neck pain -Back pain -Backache -Neckache -Dorsalgia -Thoracic -Torso -Radiculopathy -Radicular pain -Radiculitis -Disc herniation -Disk herniation -Intervertebral disc -Intervertebral disk -Intervertebral disc displacement -Intervertebral disk displacement -Disc degeneration -Disk degeneration -Spinal stenosis -Spondylolisthesis -Spondylosis -Spondylolysis -Adjacent segment disease -Junction failure -Degenerative disc disease -Degenerative disk disease -Scoliosis -Spinal osteophytosis -Neck muscles -Back muscles -Neuralgia -Whiplash injuries -Spinal injuries -Postlaminectomy -Headache -Cervical plexus -Brachial plexus -Brachialgia -Cervico-brachial neuralgia -Brachial neuritis -Brachial neuralgia -Thoracic outlet syndrome -Arthritis -Myofascial pain syndromes -Fibromyalgia -Atlanto-axial joint -Atlanto-occipital joint -Cervical rib syndrome -Polyradiculitis -Polyneuroradiculitis -Cervicogenic -Torticollis -Spondylitis -Trigger point -Spinal nerve roots -Myelopathy -Myeloradiculopathy -Radiculomyelopathy -Nerve compression syndromes

Chiropractic[tw] OR Chiropractor[tw] OR Chiropractic Adjustment[tw] OR Musculoskeletal Manipulations[tw] OR Osteopathic Manipulations[tw] OR Orthopedic Manipulations[tw] OR Manual Therapy[tw] OR Manual Therapies[tw] OR Manipulative Therapy[tw] OR Manipulative Therapies[tw] OR Manipulative Rehabilitation[tw] OR Joint Manipulation[tw] OR Joint Mobilization[tw] OR Mobilization Therapy[tw] OR Spinal Mobilization[tw] OR Spinal Manipulative Therapy[tw] OR Cervical Manipulation[tw] OR Cervical Mobilization[tw] OR Soft Tissue Mobilization[tw] OR Flexion-Distraction[tw] OR Myofascial[tw] OR Active Release[tw] OR Graston[tw] OR Massage[tw] OR Stretching Techniques[tw] OR Muscle Stretching[tw] OR Static Stretching[tw] OR Passive Stretching[tw] OR Proprioceptive Neuromuscular Facilitation[tw] OR PNF Stretching[tw] OR Post Isometric Relaxation[tw] OR Contract-Relax[tw] OR Instrument Assisted Soft Tissue[tw] OR Instrument Assisted Manipulation[tw] OR Instrument Assisted Adjustment[tw] OR Instrument Assisted Adjusting[tw] OR Manipulation Under Anesthesia[tw] OR Spinal Manipulation[tw] OR Muscle Energy Technique[tw]

### Stage 3: Study selection

#### Eligibility criteria

**Fig. 2:**
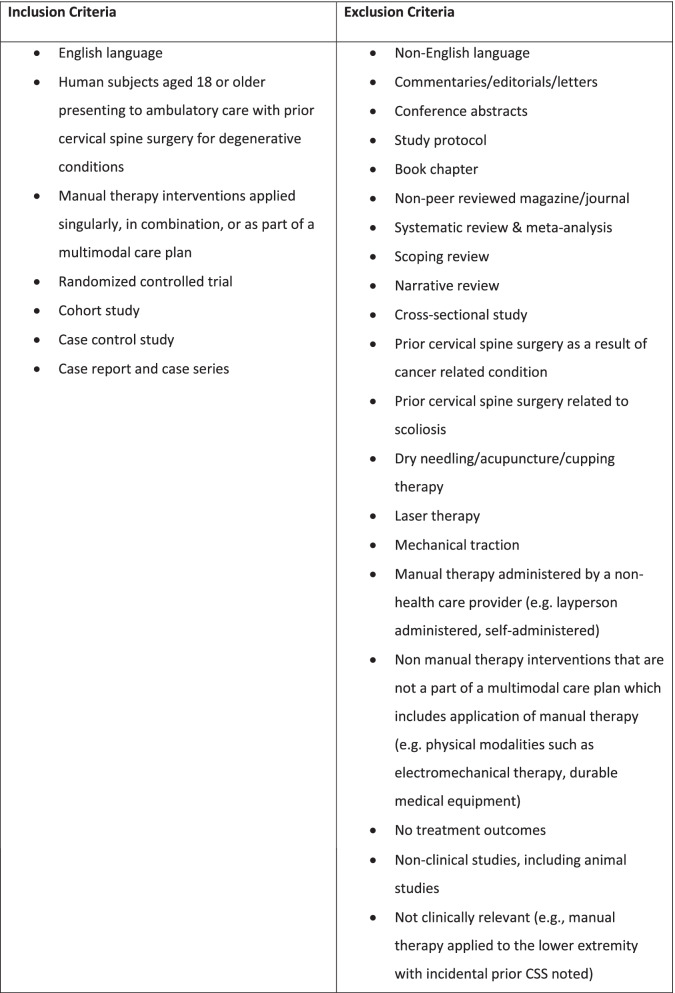
Eligibility criteria for this study

Eligibility criteria for studies in this review are listed in Fig. 2. This review focuses on English-language literature that includes quantitative and clinical observation methods in outpatient ambulatory care settings. Randomized clinical trials (RCTs), cohort studies, case–control studies, case reports and case series are included. Mixed methods studies were only considered if quantitative data could be clearly extracted. The Population, Interventions, Comparators, Outcomes (PICO) method was utilized to assist in identifying eligibility criteria.

#### Population

Studies in this review included adults (≥ 18 years of age) in ambulatory care settings with prior cervical spine surgery for degenerative conditions. Cervical spine surgeries were defined as the following surgeries performed in the cervical spine region: discectomy, laminectomy, laminotomy, foraminotomy, single-level fusion, multi-level fusion, artificial disc replacement, and spinal cord stimulator implantation. Articles which failed to provide a reason for prior cervical spine surgery are included in this review under the assumption that the surgery was due to a degenerative condition.

#### Interventions

This study targets literature that includes manual therapy interventions. For purposes of this study, manual therapy interventions are categorized into peripheral or spinal manual joint mobilization or manipulation, table or instrument assisted peripheral or spinal joint manipulation or mobilization, manually assisted musculoskeletal stretching, and soft tissue manipulation [27], and are defined in Additional file 1: File A. Articles which failed to fully describe manual therapy interventions and included common manual therapy terminology (e.g. mobilization, passive physical therapy) are included in this review under the assumption the procedures were consistent with our operationalization of manual therapy as described in Additional file 1: File A.

#### Comparators

There are no restrictions on comparator usage. Studies are eligible with or without comparison groups. There are no restrictions on composition of comparison groups, including all active treatments, placebos or shams, wait list, and no intervention.

#### Outcomes

This study considers literature with inclusion of outcomes related to pain (e.g. intensity, frequency, duration, etc.), function and objective physical examination findings (e.g. ranges of motion, activities of daily living, exercise capacity, motor strength testing, sensory testing, etc.), disability (e.g. degree of disability index, return to work, etc.), medication consumption (e.g. change in reported medication consumption, change in prescription receipt), patient satisfaction (e.g. Press-Ganey scores, patient report), and adverse events. Outcomes may be described as patient reported outcome measures (e.g. visual analogue scale, Neck Disability Index, etc.) or subjective reporting of the patient. Adverse events are described as direct or indirect [27]. Direct adverse events are any undesirable sign, symptom or disease associated with manual therapy intervention that may or may not be caused by the manual therapy intervention [27]. Indirect adverse events are any delay in diagnosis or treatment resulted from manual therapy intervention or an undesirable sign, symptom, disease, or progression of disease resulting from the delay in diagnosis or treatment [27].

#### Article selection

De-duplicated citations were uploaded from Endnote to Rayyan [28] for screening of abstracts and full texts. Paired investigators independently screened titles and abstracts for evaluation against the inclusion and exclusion criteria for eligibility (JAG, ALS). Titles and abstracts that met the review criteria after preliminary review were saved. Paired investigators then independently evaluated the full text of the selected articles to confirm inclusion in this study (JAG, ALS). Disagreement on eligibility at each stage was resolved by discussion and a third investigator review (AZD) when necessary.

### Stage 4: Charting the data

#### Data items and data extraction

Paired investigators independently extracted data from all eligible studies (JAG, ZAC, CJD, JK). Disagreement on data extraction was resolved through discussion and a third investigator review when necessary (AZD). Data items extracted were: (1) article information (e.g. citation first author and year, study design), (2) participants: demographics (e.g. age, sex), medical history (e.g. mean symptom duration, comorbidities), pre-cervical spine surgical indication/pathology (e.g. neuroforaminal/central canal stenosis, spondylolisthesis, vertebral instability, herniated nucleus pulposus, neck pain, cervical radiculopathy), cervical spine surgical history (e.g. number of cervical spine surgeries, microdiscectomy, laminectomy/laminotomy/foraminotomy, artificial disc replacement, single or multi-level fusion), post-cervical spine surgical history, (3) pre-manual therapy intervention testing/assessment (e.g. patient reported outcome measures, relevant physical examination testing/functional findings), (4) intervention and follow up (e.g. type(s) of manual therapy intervention, body region of manual therapy application, duration and dosage of care, timing of manual therapy intervention in relation to timing of cervical spine surgery, timing of follow up), (5) outcomes (e.g. pain, function, disability, medication consumption, patient satisfaction, adverse events).

#### Evaluation of risk of bias

To aid in assessing the current state of literature, evaluation of quality (risk-of-bias) of eligible articles was completed using the Scottish Intercollegiate Guideline Network (SIGN) critical appraisal checklists [29]. SIGN checklists allow investigators to assess risk-of-bias for each eligible RCT, cohort, and case–control studies. SIGN checklists score each article as “high-quality”, “acceptable”, “low-quality”, or “unacceptable”. Paired investigators independently performed quality assessment for each eligible article with study design compatible with the SIGN checklists (JAG, CJD). Disagreements were resolved with discussion and a third investigator review (AZD). Case reports and case series were not assessed for quality.

#### Strength of evidence

To further assist in the assessment of the current state of literature, evaluation of strength of evidence was performed. Strength of evidence rating was based on the quality (risk-of-bias, consistency across findings, study design) and quantity of available evidence. This assessment was determined by the authors and is a modified assessment derived from Bronfort et al. that has also been used in other review studies [15, 16, 30]. Evidence was rated as being “high-quality” if results were consistent with 2 or more high-quality (low risk-of-bias) studies. Evidence was rated as “moderate-quality” if results were consistent with one or more high-quality (low risk-of-bias) studies, 2 or more moderate-quality (acceptable risk-of-bias) studies, or there were inconsistent results with 2 or more high-quality (low risk-of-bias) studies. Lastly, evidence was rated inconclusive in the absence of studies of higher levels of hierarchical evidence (e.g. RCTs), if results from studies with higher-level hierarchical evidence (e.g. RCTs) were inconsistent with moderate-quality (acceptable risk-of-bias) studies, or if results from studies with higher-level hierarchical evidence (e.g. RCTs) were only consistent with “low-quality” (low risk-of-bias) studies.

### Stage 5: Collating, summarizing, and reporting results

Consistent with the aims of this study, results of this review were synthesized and are presented to provide meaning for clinical practice and scholarship by using a descriptive numeric summary and a qualitative thematic narrative [31].

#### Descriptive numeric summary

Characteristics of eligible studies are described, such as number of studies included, types of study design, medical history of subjects, pre-surgical pathology/indication and cervical spine surgeries, manual therapy interventions, pre-intervention assessments, post-intervention outcomes, and adverse events. Quality (risk-of-bias) assessment is also described for each eligible RCT, cohort, and case–control study.

#### Qualitative thematic narrative

Based on the findings of the eligible studies in this review, a qualitative thematic narrative is organized by surgical type (e.g. fusion, discectomy, disc replacement) and manual therapy intervention type (e.g. joint mobilization or manipulation, table or instrument assisted mobilization or manipulation, manual therapy not otherwise classified, multimodal approaches along with manual therapy). A description of literature informing the rating of the strength of evidence is included for each thematic group.

## Results

### Descriptive numerical summary

The study selection process is illustrated in the flow diagram (Fig. 1). Articles that were excluded at the full-text review stage are listed with reasons for exclusion in Additional file 2: File B.

Key findings from the eligible studies in this review are described in Tables 2, 3 and 4. A descriptive report of included studies, which includes study design, patient demographics, medical history, surgical history, post-surgical history, and adverse events are shown in Table 2. Descriptions of manual therapy interventions and outcomes are shown in Table 3. An overview of surgical type, manual therapy type, and the reporting of adverse events are shown in Table 4. Quality (risk-of-bias) assessment for the 2 included RCTs are shown in Table 5.

**Table 2:** Descriptive report of included studies

Citation	Study design (n)	Years of age	Sex	Medical history	Pre-surgical pathology/ indication	Surgical history	Post-surgical history	Adverse events
Casagrande et al. [34]	Case report (1)	29	Male	Unknown cause of initial onset of neck and right shoulder pain with limited mobility due to lack of strength and pain Failed nonoperative therapy prior to CSS	Weakness in right arm abduction No biceps reflex MRI revealed right sided C4-C5 HNP compressing 5th nerve root	Right-sided anterior discectomy and interbody fusion with autologous bone from left iliac crest, plate placement between C4-C5	No surgical complications, discharged without pain Advised to wear Philadelphia cervical collar for 4-weeks 4-weeks post-operative x-rays revealed no abnormalities 10-weeks post-operative CT revealed no abnormalities and “good fusion” between C4-C5	Not reported
Cole et al. [49]	Case report (1)	70	Male	Presented to chiropractic clinic with chronic radiating LBP and cervical / thoracic junction pain Alcohol dependence in remission, PTSD and depression previously requiring hospitalization Lumbar laminectomy Long-term opioid therapy	Not Reported	C3-C7 fusion	Chronic cervical / thoracic junction pain Prior course of physical therapy, interventional spine procedures, long-term opioid therapy	Not Reported (Response to care following initial visit was reported to be without adverse effects)
Cooper and Golberg [35]	Case report (1)	43	Female	Extensive history of neck pain	Not reported	C6-C7 anterior fusion	Diagnosed with acquired cervical kyphosis, with associated cervicalgia, thoracic spine pain, lumbago	Not reported
Harrison et al. [36]	Case report (1)	62	Male	Not reported	C5-C6 instability, vertebral spondylosis, HNP	C5-C6 fusion using autologous iliac crest bone graft (13-years prior to intervention) 2^nd^ operation consisting of anterior fusion with plate and autologous bone (12-years prior to intervention)	Patient continued to suffer from post-surgical axial and radicular symptoms Patient sought treatment for neck pain, numbness, tingling in right anterolateral forearm, and right arm weakness	Not reported
Murphy and Morris [37]	Case Report (1)	52	Male	Acetaminophen and oxycodone provided relief of neck pain ROS: recent onset of bilateral tinnitus; occasional chills and “fevers”; new onset balance problems; history of smoking and ETOH consumption; no regular exercise BP 155/90; Temperature 97.5 Fahrenheit (36.3 Celcius); respirations 25/minute; pulse rate 102 bpm	Not reported	C5-C6, C6-C7 anterior fusion (8 years prior to intervention) 2^nd^ operation with insertion of instrumentation (6 years prior to intervention)	Recurrent episodes of neck pain Presented to ED 1 week prior to intervention for sharp pain in lower cervical area with referral to left shoulder; given a soft collar and released to follow up with PCP PCP referred patient for chiropractic evaluation	Mortality
Polkinghorn and Colloca [38]	Case report (1)	35	Female	15-year history of neck pain and cervical muscle spasm 6-month failed course of analgesics, NSAIDs, PT	Not reported	C3-C4 discectomy C5-C6 fusion 6-months following 1st surgery	Pain persisted after 2nd surgery for another 12-months Episodic cervical muscle spasms Condition exacerbated by cold/damp weather	Not reported
Salvatori et al. [39]	Case report (1)	46	Female	Osteoarthritis, HTN, LBP, neck pain with headache	1-year history of neck pain, headaches, frequent fatigue of upper quarter, intermittent pain referred to LUE	C5-C6, C6-C7 ACDF (8-weeks prior to intervention)	6-weeks immobilization of cervical spine with Aspen collar Improved pain referral to LUE No improvement in headache frequency or intensity, neck pain, upper quarter fatigue New onset of restricted cervical flexion and extension ROM, cervical muscle tightness and fatigue, intermittent referred pain to RUE	Not reported
Tibbles [42]	Case report (1)	28	Male	Initial onset of neck and upper back pain secondary to carrying daughter on shoulders; 24 h later experienced RUE numbness 4 1/2-month subsequent history of neck pain with radiation into RUE prior to CSS	C6–C7 right posterolateral HNP	C5–C6 discectomy	Persistent arm pain at discharge 6-weeks post-operative CT revealed C6-C7 HNP, surgical intervention completed at incorrect cervical (C5-C6) level Lower right-sided neck pain radiating into right trapezius muscle	Not reported
Bloink and Blum [43]	Case report (2)	30 52	Male Female	Ski related injury; unable to run/walk > 1/2 mile due to pain Use of dental device Not reported	Loss of sensation, function of right 3rd and 4th fingers; 5 months of physical therapy without improvement Significant neck pain with pain radiating into right arm and 2nd, 3rd fingers	C5-C6 disc replacement C5–C6, C6–C7 disc replacement	Symptoms improved for 3 months with recurrence of right neck pain, periscapular, and upper arm pain; experienced same symptoms on left side 2 x/week 3-months post-operative cervical MRI negative for pathology; attended physical therapy without improvement, trialed Neurontin Symptoms resolved initially with recurrence and progressive worsening in right arm; developed left arm to finger pain	Not Reported Not reported
Malone et al. [40]	Case series (2)	59 49	Male Male	Chronic neck pain Not reported	C7 right radiculopathy Not reported	C6-C7 allograft ACDF C4-C5 fusion	Not reported Fell at work, developed hand tingling and neck pain which he sought cervical SMT	Loss of function in hands followed by loss of ability to ambulate; decrease in UE strength; broad and spastic gait; diminished lower extremity proprioception; MRI revealed C5-C6 HNP causing marked spinal cord compression and abnormal signal in cord; underwent C6 surgical corpectomy and allograft strut- and plate-assisted fusion Worsening of right arm pain and weakness; diminished grip strength; 3 + DTRs; positive Hoffman bilaterally; radiography revealed HNP compressing cord at C5-C6; surgical intervention resulted
Peolsson et al. [32]	Randomized Clinical Trial (63)	Mean age 46	34 men, 29 women	Inclusion Criteria: 18–65 years of age Cervical radiculopathy for ≥ 8-weeks but < 5-years	MRI with confirmed nerve root compression due to CDD of 1 or 2 segmental levels	Group 1: ACDF included in intervention Group 2: No prior CSS	Not applicable	Not reported
Ren et al. [33]	Randomized Clinical Trial (86)	Mean age 54.2	29 men, 43 women	Inclusion Criteria: > 18 years of age Anxiety disorder ≥ 6 months prior to surgery > 1-day post-operative following open reduction and internal fixation surgery	Not Reported	Group 1: Open reduction and internal fixation Group 2: Open reduction and internal fixation	Not applicable	Not reported

*CSS* cervical spine surgery, *MRI* magnetic resonance imaging, *CT* computed tomography, *PTSD* post-traumatic stress disorder, *HNP* herniated nucleus pulposus, *ROS* review of systems, *ETOH* alcohol, *ED* emergency department, *PCP* primary care provider, *NSAIDs* non-steroidal anti-inflammatory drugs, *PT* physical therapy, *HTN* hypertension, *LBP* low back pain, *LUE* left upper extremity, *ACDF* anterior cervical discectomy and fusion, *ROM* ranges of motion, *RUE* right upper extremity, *SMT* spinal manipulative therapy, *CDD* cervical degenerative disease

**Table 3:** Intervention description and outcomes

Citation	Pre-intervention assessment/testing	Intervention	Length of intervention	Longitudinal follow-up	Clinical outcomes	Patient satisfaction
Casagrande et al. [34]	Not reported	After 4-weeks of rest the patient started a rehabilitation program 2-weeks of Tecar Therapy sessions, manual passive physical therapy, deltoid muscle electrostimulation After 2-weeks, 2 × /week of hydrokinesis sessions, hydrobike, walking, water walking, running After 8-weeks restart working directly on soccer field	8-weeks	Playing professional soccer (“Serie B”) 5-years post-operative	Return to sport (work) after less than 4-months	Not reported
Cole et al. [49]	10 mg hydrocodone, 3–4 × daily Average NRS 6/10 Best NRS 4/10 Worst NRS 10/10 BBQ 48/70	7 visits: Myofascial release to thoracic and lumbar musculature HVLA SMT to cervicothoracic junction and thoracic spine Table-assisted drop SMT to sacroiliac joints Table-assisted flexion distraction SMT Home care consisting of stretching, foam rolling, end range loading	Undetermined (at least 3 months duration)	1-week, 2-month follow ups, undetermined thereafter	Opioid therapy discontinued NRS 3/10 BBQ 30/70	Not reported
Cooper and Golberg [35]	Not reported	Patient presented 9 × just over 1-month with 6 SMT, 2 of which were cervical Cervical SMT consisted of consecutive T1, T2 prone toggle table assisted thrust; C5 instrument assisted thrust using 25 pounds of force	~ 1-month	Not reported	Patient reported “significant” pain reduction	Not reported
Harrison et al. [36]	Patient reported condition interfered with work duties Right-sided weakness in grip strength NRS 6/10 NDI 18% disability ROM: 32◦ Ext 48◦ Flex 23◦ L Rot 69◦ R Rot 31◦ L LF 27◦ R LF	10 visits over ~ 1-month: Manual and instrument assisted SMT to non-fused cervical and upper thoracic spine Cervical rotational stretching Cervical and thoracic myofascial therapy Cervical and thoracic region cryotherapy 11 visits over ~ 1-month: “Mirror image postural” SMT Manual and instrument assisted SMT to cervical and thoracic spine Mirror image exercise Mirror image cervical spine extension traction 8 visits over 4-months: Combination of above treatments 30 visits over 26-months: Combination of above treatments 59 total visits	~ 32-months	1, 2, 6, 21, 32-month follow ups	1-month follow up: Decreased C5–C6 dermatome sensation Right-sided weakness in grip strength NRS 2/10 NDI 22% disability ROM: 32◦ Ext 50◦ Flex 27◦ L Rot 59◦ R Rot 27◦ L LF 23◦ R LF 2-month follow up: NRS 1/10 NDI 12% disability ROM: 44◦ Ext 50◦ Flex 23◦ L Rot 63◦ R Rot 28◦ L LF 34◦ R LF 6-month follow up: NRS 1/10 NDI 10% disability 21-month follow up: Normal C5-C6 dermatome sensation Patient reported perceived increased grip strength Patient returned to work 32-month follow up: NRS 2/10 NDI 8% disability	Not reported
Murphy and Morris [37]	Motor strength was + 5/5 bilaterally throughout DTRs were absent with the exception of ankle jerks (1 + bilaterally and symmetric) ROM of cervical spine was restricted and painful in all directions	Initial recommendation to apply ice to cervical spine and maintain mobility Patient returned the following day: Administered C2-C3 SMT using lateral flexion muscle energy technique with patient in supine posture with instruction to continue ice application at home Patient returned the next day reporting inability to lift left arm and a “buzzing” sensation throughout the thoracic, lumbar regions MRI was performed the following day	2 days	Not Applicable	Patient died of heart failure while receiving MRI MRI revealed epidural abscess extending from C2-C4 within right posterior epidural space	Not reported
Polkinghorn and Colloca [38]	Unable to demonstrate cervical ROM due to pain Psychologically distraught	Instrument assisted cervical SMT	Total of 30 treatments over 8-months; initially 3x/week with progressive decrease in treatment frequency	1-week, 1-month, 2-month, 2-year follows ups	End of week 1, acute exacerbation resolved After 1 month almost all previous chronic neck pain resolved After 2 months patient was pain-free and observable cervical range of motion had improved to near normal; Patient resumed strenuous physical activity (skiing, jogging, and vigorous exercise) At 2-years chronic neck problem completely resolved	Patient reported satisfaction
Salvatori et al. [39]	NPRS neck: 10 NPRS headache: 3 NDI: 46 Cervical ROM: 30◦ Ext 18◦ Flex 25◦ L Rot 10◦ R Rot 10◦ L LF 15◦ R LF Grip strength (kg): Left 22.7 Right 22.2 DNF Endurance Test: 3	ROM—therapeutic exercise interventions included active cervical rotation, Flex and Ext self-mobilization techniques for thoracic spine Strength was addressed using a gradual progression from cervical isometric exercises, supine DNF exercises, to isotonic cervical exercises and a combination of cervical and thoracic spine postural strengthening during functional positions Therapeutic exercises were progressed from an emphasis on increasing mobility, followed by exercises dosed for endurance and strength At the 2nd visit, thoracic spine thrust SMT was initiated	12 physical therapy sessions over 6 weeks	6 weeks	NPRS neck: 0 NPRS headache: 0 NDI: 16 Cervical ROM: 62◦ Ext 65◦ Flex 70◦ L Rot 75◦ R Rot 35◦ L LF 33◦ R LF Grip strength (kg): Left 29.5 Right 35.4 DNF Endurance Test: > 90	Not reported
Tibbles [42]	Decreased ROM and pain with cervical ext and r rot Decreased C6 dermatome to light touch on right + 4/5 strength right biceps 1 cm wasting in right biceps	Gentle cervical SMT at C5-C6 level on painful side	1.5 weeks	1.5 weeks, 4.5 weeks	Felt 80% better after 1.5 weeks of treatment—only slight neck pain, occasional numbness in arm 4.5 weeks after beginning treatment—pain free with slight right wrist extensor muscle weakness (4 + /5)	Not reported
Bloink and Blum [43]	Unable to run/walk > 1/2 mile Strength: + 4/5 right supraspinatus, + 4/5 right infraspinatus, + 4/5 right subscapularis, + 4/5 right teres minor, + 4/5 right triceps, + 4/5 bilateral deltoids NRS 8–9/10 Cervical ROM: Bilateral Rotation 10 degrees with pain Cervical Flexion, Extension, Bilateral Lateral Flexion produced neck pain Strength: + 4/5 bilateral supraspinatus, + 4/5 bilateral infraspinatus, + 4/5 bilateral deltoids + 4/5 right subscapularis, + 4/5 right teres minor, + 4/5 right triceps, + 4/5 right biceps	12 visits over ~ 2 months: Category 1 SOT blocking, intra-oral cranial adjustments, sphenomaxillary cranial treatment Immediate co-management with dental office 10 visits over ~ 5 weeks consisting of category 1 SOT blocking, intra-oral cranial adjustments, sphenomaxillary cranial treatment; 3 of these visits included immediate co-management with dental office 14 visits over ~ 16 weeks consisting of treatment of the thoracic, lumbar, sacroiliac regions	~ 2-months ~ 21-weeks	~ 2-month ~ 5, 21-week follow-ups	Hiked 10 miles which he reported he had not been able to for 2 1/2 years Ran one mile without experiencing any symptoms Cervical spine and arm pain abolished with occasional right periscapular pain Cervical spine ROM returned to normal in all directions ~ 5-week follow up: NRS 3/10 during provocative activities Significantly reduced right upper extremity pain Left arm symptoms resolved 5 + upper extremity strength throughout ~ 21-week follow up: Occasional pain in right shoulder and bicep occurring after participating in strenuous activities	Not Reported Not Reported
Malone et al. [40]	Not reported Not reported	Series of neck SMT of unknown quantity or duration Cervical SMT	Not reported Not reported	Not reported Not reported	Not reported Not reported	Not reported Not reported
Peolsson et al. [32]	VAS neck (0–100 mm) VAS arm (0–100 mm) NDI Neck ROM Hand strength NME Manual Dexterity Arm Elevation	Group 1: ACDF with postoperative PT (n = 31) Post-operative advice including ROM, posture, ergonomics, and avoiding static workload 6-weeks post-operative PT same as group 2 Group 2: PT alone (n = 32) Structured program with gradual progression through defined set of exercises integrated with cognitive-behavioral approach Medical exercise therapy focused on neck stabilization and endurance, strengthening of scapular muscles, stretching neck and shoulder muscles, thoracic mobilization Program was performed 2x/week for 14 weeks Education in pain management was conducted 1/week for 14 weeks 18 patients who experienced dizziness were also instructed in vestibular rehabilitation	14 weeks	6, 12, 24-month follow ups	No significant differences in any reported outcome measures between groups	Not reported
Ren et al. [33]	Neck pain VAS NDI Self-Rating Anxiety Scale QUALEFFO-41	Group 1: Routine Care and Foot Massage (n = 43) Routine care (undefined) and 10-min foot massage every other day for 4 weeks, starting 2-days post-operative Group 2: Routine Care Only (n = 43) Routine care undefined	4 weeks	4 week follow up	No significant difference between groups for neck pain VAS and NDI Intervention demonstrate significant improvement in Self Rating Anxiety Scale compared to pre-test and to control group The pain subscale of the quality of life scale was significantly improved for pain compared to control and only the intervention group showed significant improvement in mental function	Not reported

*BBQ* back beliefs questionnaire, *SMT* spinal manipulative therapy, *NRS* numeric [pain] rating scale, *NDI* neck disability index, *ROM* ranges of motion, *Ext* extension, *Flex* flexion, *L Rot* left rotation, *R Rot* right rotation, *L LF* left lateral flexion, *R LF* right lateral flexion, *DTRs* deep tendon reflexes, *BP* blood pressure, *bpm* beats per minute, *MRI* magnetic resonance imaging, *NPRS* numerical pain rating scale, *kg* Kilograms, *DNF* deep neck flexors, *cm* centimeters, *UE* upper extremity, *HNP* herniated nucleus pulposus, *VAS* visual analogue scale, *NDI* neck disability index, *NME* neck muscle endurance, *ACDF* anterior cervical discectomy and fusion, *PT* physical therapy, *QUALEFFO-41* quality of life questionnaire for patients with osteoporosis vertebral fractures

**Table 4:** Summary of surgical type, manual therapy interventions, and adverse events

Citation	Surgical intervention (years prior to manual therapy intervention)	Manual therapy applied to cervical region	Manual therapy applied to thoracic region	Manual spinal mobilization or manipulation	Table assisted mobilization or manipulation	Instrument assisted joint manipulation or mobilization	Manual therapy intervention(s) not otherwise classified	Multimodal approach combining manual therapy with other intervention(s)	Adverse event reported
Bloink and Blum [43]	C5-C6 Disc Replacement C5-C6, C6-C7 Disc Replacement						X X	X X	
Casagrande et al. [34]	C4-C5 ACDF (4 weeks)						X	X	
Cole et al. [49]	C3-C7 Fusion	X		X	X			X	
Cooper and Golberg [35]	C6-C7 Anterior Fusion	X	X		X	X			
Harrison et al. [36]	C5-C6 Anterior Fusion (13 years) C5-C6 Anterior Fusion With Plate (12 years)	X	X	X		X	X	X	
Murphy and Morris [37]	C5-C7 Anterior Fusion (8 years) C5-C7 Anterior Fusion With Instrumentation (6 years)	X		X				X	X
Polkinghorn and Colloca [38]	C3-C4 Discectomy C5-C6 Fusion	X				X			
Salvatori et al. [39]	C5-C7 ACDF		X				X	X	
Tibbles [42]	C5-C6 Discectomy	X					X		
Malone et al. [40]	C6-C7 ACDF C4-C5 Fusion	X X					X X		X X
Peolsson et al. [32]	ACDF (6 weeks)		X				X	X	
Ren et al. [33]	Open reduction, internal fixation						X	X

**Table 5:** Quality (Risk-of-bias) assessment of included RCT

First author and year published	Items on SIGN checklist										
	1	2	3	4	5	6	7	8	9	10	Quality
Peolsson et al. [32]	Y	Y	CS	N	CS	CS	CS	CS	CS	CS	L
Ren et al. [33]	Y	Y	Y	N	Y	Y	Y	N	CS	NA	A

Y = Yes, N = No, CS = Cannot say, NA = Not applicable

Quality: H = High, A = Acceptable, L = Low

SIGN, Scottish Intercollegiate Guideline Network

Quality assessment items from checklist:

1. Study addresses an appropriate and focused question

2. Assignment of subjects to treatment groups is randomized

3. An appropriate concealment method is used

4. Subjects and investigators are blind to treatment allocation

5. Treatment and control groups are comparable at start of trial

6. Only difference between groups is treatment under investigation

7. Relevant outcomes are measured using standard, valid, and reliable methods

8. Percentage (%) of dropout

9. Subjects are analyzed in the groups which they were randomly allocated (intention-to-treat analysis)

10. If study utilizes > 1 site, results are comparable across all sites

### Qualitative thematic narrative

#### Fusion

There was 1 RCT of low-quality with 63 participants and 1 RCT of acceptable-quality with 86 participants which met inclusion criteria [32, 33]. Peolsson et al. [32] investigated ACDF with postoperative structured physical therapy that included thoracic mobilization compared to structured physical therapy without ACDF for individuals with cervical radiculopathy. Ren et al. [33] investigated the effects of foot massage on relieving pain, anxiety, and quality of life among patients that have undergone a cervical open reduction and internal fixation surgery.

A total of 8 case reports or series were identified describing 9 patients with history of cervical spine fusion surgery [34–41]. Favorable clinical outcomes encompassing return to work (sport) [34, 36], pain reduction [35, 36, 38, 39, 41], increased cervical ranges of motion [36, 38, 39], improved disability index [36, 39], improved fear reduction [41], increased sensation [36], increased grip strength [36, 39], increased deep neck flexor muscle endurance [39], increased physical activity [38], and reduction of opioid therapy [41] were described in 6 patients across 6 case reports [34–36, 38, 39, 41].

Adverse events were reported in 3 patients across 2 case studies [37, 40]. Murphy et al. [37] described mortality in a 52-year old male. A magnetic resonance imaging (MRI) study was performed on the fourth day after initial chiropractic evaluation which revealed an epidural abscess within the right posterior epidural space extending from C2 to C4. The patient died of heart failure during the MRI examination. Malone et al. [40] described two cases of complications that occurred after reported cervical spine manipulation that resulted in surgical intervention.

There is currently a lack of quality (low and moderate risk-of-bias) studies of higher-level hierarchical study designs to inform evidence related to clinical outcomes, patient satisfaction, and adverse events associated with manual therapy for patients with prior cervical fusion surgery due to degenerative conditions.

#### Discectomy

There were 2 case reports describing a total of 2 patients with a history of cervical discectomy [38, 42]. Favorable outcomes were described to include decreased pain [38, 42], increased cervical ranges of motion [38], increase in physical activity [38], and satisfaction with care [38]. No adverse events were reported.

There is currently a lack of studies with higher-level hierarchical study designs to inform evidence on clinical outcomes, patient satisfaction, and adverse events associated with manual therapy for patients with prior cervical spine discectomy surgery due to degenerative conditions is rated as inconclusive due to a lack of study design of higher-level hierarchical evidence.

#### Disc replacement

There was 1 case report which included 2 patients with cervical disc replacement surgery [43]. Favorable clinical outcomes included increase in physical function, decrease in pain, and increase in cervical ranges of motion. No adverse events were reported.

There is currently a lack of studies with higher-level hierarchical study design informing evidence related to clinical outcomes, patient satisfaction, and adverse events associated with manual therapy for patients with prior cervical disc replacement surgery due to degenerative conditions.

#### Manual spinal joint mobilization/manipulation

Manual spinal joint mobilization or manipulation was described in 3 studies involving 3 patients [36, 37, 41]. Favorable clinical outcomes were seen in 2 patients in 2 case reports and included return to work, pain reduction [36, 41], increased cervical ranges of motion [36], decreased disability index [36], increased sensation [36], increased grip strength [36], improvement in fear reduction [41], and reduction of opioid therapy [41]. There was no reporting of patient satisfaction in cases that described the use of manual joint mobilization or manipulation. One case described mortality due to heart failure in a patient with a cervical epidural abscess [37].

There is currently a lack of studies of higher-level hierarchical study design informing evidence related to clinical outcomes, patient satisfaction, and adverse events associated with manual joint mobilization or manipulation for patients with prior cervical spine surgery due to degenerative conditions.

#### Table/instrument assisted spinal joint mobilization/manipulation

Table or instrument assisted spinal joint mobilization or manipulation was described in 4 case reports involving 4 patients [35, 36, 38, 41]. Favorable clinical outcomes were seen in all 4 patients across all 4 studies and included return to work [36], pain reduction [35, 36, 38, 41], increase in cervical ranges of motion [36, 38], decreased disability index [36], increased sensation [36], increased grip strength [36], increased physical activity [38], decrease in fear avoidance [41], and reduction of opioid therapy [41]. One patient reported satisfaction [38] and there were no adverse events reported.

There is currently a lack of studies of higher-level hierarchical study design informing evidence related to clinical outcomes, patient satisfaction, and adverse events associated with table or instrument assisted joint mobilization or manipulation for patients with prior cervical spine surgery due to degenerative conditions.

#### Manual therapy interventions not otherwise classified

Use of manual therapy interventions that are not otherwise classified in this review were described in 6 case reports/series involving 8 patients and 2 RCTs involving 149 patients [32–34, 36, 40, 42, 43]. Favorable clinical outcomes were seen in return to (sport) work [34, 36], pain reduction [36, 42, 43], increase in cervical ranges of motion [36], improvement in NDI scores [36], increased strength [36, 43], and increased physical activity [43]. Adverse events were described in 1 case series involving 2 patients which required surgical intervention [40]. Patient satisfaction was not reported.

There is currently a lack of quality (low and moderate risk-of-bias) studies of higher-level hierarchical study designs to inform evidence related to clinical outcomes, patient satisfaction, and adverse events, associated with manual therapy that is not otherwise classified in this review for patients with prior cervical fusion surgery due to degenerative.

#### Multimodal approach combining manual therapies with other interventions

The use of multimodal approaches that included manual therapy along with other forms of intervention were described in 6 case reports/series involving 7 patients, and 2 RCTs involving 149 patients [32–34, 36, 37, 39, 41, 43]. Favorable clinical outcomes were seen in return to (sport) work [34, 36], pain reduction [36, 39, 41, 43], increase in cervical ranges of motion [36, 39, 43], improvement in NDI scores [36, 39], increased strength [36, 39, 43], increase in cervical deep neck flexor muscular endurance [39], improvement in fear reduction [41], increase in physical activity [43], and reduction in opioid therapy [41]. One case described mortality secondary to heart failure in a patient with a cervical epidural abscess [37]. There was no reporting of patient satisfaction.

There is currently a lack of quality (low and moderate risk-of-bias) studies of higher-level hierarchical study designs to inform evidence related to clinical outcomes, patient satisfaction, and adverse events, associated with use of multimodal interventions along with manual therapy for patients with prior cervical fusion surgery due to degenerative.

## Discussion

The current state of literature on manual therapy for individuals with prior cervical spine surgery for degenerative conditions is in its infancy. This scoping review identified 12 articles that met eligibility criteria with 8 of the 12 articles published since 2013 [32–36, 39, 41] and the oldest article published approximately 30 years ago (1992) [42]. The literature is almost exclusively comprised of low-level studies with 10 of 12 eligible studies consisting of case reports or series [34–42]. There was 1 low-quality RCT and 1 acceptable-quality RCT identified in the literature [32, 33].

This review reinforces the presence of manual therapy intervention administered to patients with history of cervical spine surgery that is seen in clinical practice. Evidence associated with clinical outcomes for manual therapy for this population was unable to be ascertained. Multiple articles in this review described a favorable clinical response to care, however the literature cannot currently provide clinical guidance due to the limitations of study design and quality. Moreover, although multiple cervical spine surgical procedures are routinely completed, the literature is only representative of administration of manual therapies in individuals with prior cervical fusion, cervical discectomy, and cervical disc replacement surgeries. Similarly, a variety of manual therapy interventions are commonly administered in clinical practice, yet literature does not currently contain a robust number of studies on any one type of manual therapy intervention; further contributing to the uncertainty, most studies fail to fully describe the scope and techniques of the manual therapy interventions reported in the article [32–34, 36, 39–42].

The impact of patient satisfaction in clinical care is not fully known and the relationship between patient satisfaction, outcomes, and costs are questionable [44]. Nevertheless, patient satisfaction is an increasing component of health care delivery assessment. Only one study in this review included reporting of patient satisfaction [38]; unfortunately, the description provided in this article was vague and failed to utilize standardized patient satisfaction instruments, such as Press-Ganey scores [45]. Future studies involving manual therapy interventions in individuals with prior cervical spine surgery should include assessment of patient satisfaction metrics and investigate the relationship between satisfaction, outcomes, and costs.

Adverse events associated with manual therapy to the spine are most commonly benign and transient in nature [46]. Serious adverse events are less common and are considered rare [46]. This review identified 2 studies describing 3 total patients with serious adverse events that occurred after manual therapy interventions [37, 40]. Two patients underwent surgical intervention for neurologic deficit and cord compression due to a herniated spinal disc [40] and one patient died due to heart failure during a MRI which revealed an abscess in the cervical epidural space [37]. Unfortunately, literature available in this review does not allow for an adequate assessment of associations between manual therapy and adverse events. The number of adverse events reported may initially appear as an alarmingly high proportion compared to our overall sample in this review. However, this may be due to clinicians being more likely to report on adverse events versus a potentially inconsequential treatment outcome. Further, based on the potential of complexities of comorbid factors such as time sensitive challenges in diagnosis for a condition such as epidural abscess and the condition’s natural history [37, 47], limited historical accounting [40], and the nature of the studies’ design [37, 40] no causal association between manual therapy intervention and adverse events can be determined. Nonetheless, cases reporting adverse events are important to consider for future study to explore the prevalence and potential association between clinical interventions and adverse events so that safety profiles and risk–benefit assessments can be established.

## Strengths and limitations

This review has important strengths and implications. A methodologically rigorous review was completed, adhering to recommended frameworks [20–22], and was conducted by a team with experienced researchers and health science librarians. To our knowledge, this is the first scoping review to identify and describe manual therapy interventions, associated outcomes, and adverse events reported for individuals with a history of cervical spine surgery. This review illustrates the gap in this body of knowledge and emphasizes the need for higher-level studies of high-quality to allow for recommendations on manual therapy interventions in the management of adults with prior cervical spine surgery. It is expected this review will lead to further interest and opportunities to complete high-quality clinical research in this field. This study had 3 notable limitations. First, this study was a scoping review which is subject to inconsistent definition and methodology which may pose difficulty in comparison of results in future reviews [48]. Second, though we had no exclusions due to language in our search, our data extraction of identified eligible articles was limited to the English-language. There is potential this review failed to include relevant studies outside of the English-language. Third, this review was comprised mostly of studies of very low hierarchical evidence and therefore conclusions on outcomes and adverse events cannot be inferred.

## Conclusions

Following cervical spine surgery for degenerative conditions, there is a dearth of literature that is currently available and is limited to case reports, case series, and 2 RCTs. Given that manual therapy is currently being applied to individuals with prior cervical spine surgery due to degenerative conditions, future research is needed to examine the clinical utility and safety profile to support evidenced-based clinical practice.

## Supplementary Information

**Additional file 1:** Definition and examples of manual therapy interventions.

**Additional file 2:** Excluded citations.

## Data Availability

All data collected are included and described in this manuscript.

## Abbreviations

ACDF: Anterior cervical discectomy and fusion
PRISMA-ScR: Preferred reporting items for systematic reviews and meta-analyses extension for scoping reviews
CINAHL: Cumulative index of nursing and allied health literature
PEDro: Physiotherapy evidence database
WHO: World Health Organization
RCT: Randomized clinical trial
PICO: Population, interventions, comparators, outcomes
SIGN: Scottish intercollegiate guideline network
MRI: Magnetic resonance imaging
