# Supplementary material for: Manual therapy interventions in the management of adults with prior cervical spine surgery for degenerative conditions: a scoping review
Source: Chiropr Man Therap. 2022 Mar 7;30:13. doi: 10.1186/s12998-022-00422-8 (PMC8900329; doi:10.1186/s12998-022-00422-8)
Supplement: Supplementary file 1 — Additional file 1: Definition and examples of manual therapy interventions. [file 12998_2022_422_MOESM1_ESM.docx]

Supplementary File A. Definition and examples of manual therapy interventions.

| **Manual Therapy Intervention** | **Definition** | **Examples** |
| --- | --- | --- |
| Joint Mobilization | Application of a low-velocity and minimal or large amplitude oscillatory motion within a joint’s passive range of motion^£^ | - Peripheral joint mobilization - Cervical spine mobilization - Thoracic spine mobilization |
| Joint Manipulation | Application of a high velocity and low amplitude thrust targeted at or near the joint’s passive range of motion^£^ | - Peripheral joint manipulation - Cervical spine manipulation - Thoracic spine manipulation |
| Manually assisted musculoskeletal stretching | Manually assisted active or passive range of motion stretching with or without soft tissue manipulation | - Passive range of motion stretching - Strain/counterstrain stretching - Manually assisted nerve flossing maneuvers |
| Manual or instrument assisted soft tissue manipulation | Application of a manual or instrumental pressure to the soft tissue structures^£^ | - Myofascial release techniques - Instrument assisted soft tissue manipulation (IASTM) techniques - Massage - Muscle energy technique |

^£^Cancelliere C, Wong JJ, Yu H, et al. Postsurgical rehabilitation for adults with low back pain with or without radiculopathy who were treated surgically: protocol for a mixed studies systematic review. *BMJ Open*. 2020;10(3):e036817. doi:10.1136/bmjopen-2020-036817
