# Supplementary material for: Manual therapy interventions in the management of adults with prior cervical spine surgery for degenerative conditions: a scoping review
Source: Chiropr Man Therap. 2022 Mar 7;30:13. doi: 10.1186/s12998-022-00422-8 (PMC8900329; doi:10.1186/s12998-022-00422-8)
Supplement: Supplementary file 2 — Additional file 2: Excluded citations. [file 12998_2022_422_MOESM2_ESM.docx]

Supplementary File B. Excluded citations.

**No prior cervical spine surgery or surgery due to trauma (122)**

1. Abd Jalil N, Awang MS, Omar M: Scalene myofascial pain syndrome mimicking cervical disc prolapse: a report of two cases. Malays J Med Sci 17:60-66, 2010

2. Abdo WF, Bloem BR, Eijk JJ, Geurts AC, van Alfen N, van de Warrenburg BP: Atypical dystonic shoulder movements following neuralgic amyotrophy. Movement Disorders 24:293-296, 2009

3. Abe T, Nagamine Y, Ishimatsu S, Tokuda Y: Spinal epidural hematoma after stretch exercise: a case report. American Journal of Emergency Medicine 27:902.e901-902, 2009

4. Aksoy IA, Schrader SL, Ali MS, Borovansky JA, Ross MA: Spinal accessory neuropathy associated with deep tissue massage: a case report. Archives of Physical Medicine & Rehabilitation 90:1969-1972, 2009

5. Alai NN, Skinner HB, Nabili ST, Jeffes E, Shahrokni S, Saemi AM: Notalgia paresthetica associated with cervical spinal stenosis and cervicothoracic disk disease at C4 through C7. Cutis 85:77-81, 2010

6. Albuquerque FC, Hu YC, Dashti SR, Abla AA, Clark JC, Alkire B, et al: Craniocervical arterial dissections as sequelae of chiropractic manipulation: patterns of injury and management. J Neurosurg 115:1197-1205, 2011

7. Alcantara J, Plaugher G, Thornton RE, Salem C: Chiropractic care of a patient with vertebral subluxations and unsuccessful surgery of the cervical spine. Journal of Manipulative & Physiological Therapeutics 24:477-482, 2001

8. Alimi YS, Di Mauro P, Fiacre E, Magnan J, Juhan C: Blunt injury to the internal carotid artery at the base of the skull: six cases of venous graft restoration. J Vasc Surg 24:249-257, 1996

9. Almeida GPLo, Carneiro KKA, Marques AlP: Manual therapy and therapeutic exercise in patient with symptomatic cervical spondylotic myelopathy: A case report. Journal of Bodywork & Movement Therapies 17:504-509, 2013

10. Bates WT: POSTOPERATIVE DISK MANAGEMENT IN SPORTS. Phys Ther 44:997-998, 1964

11. Benningfield RC: Conservative treatment and back-strengthening exercises to prevent recurrent surgery: A case report. J Sports Chiropr & Rehabil 11:52-56, 1997

12. Berner N, Capoferri D: Case study. Complete cervical kyphosis correction and resolution of low back pain utilizing Pierce Technique [case report]. Ann Vert Sublux Res 2011:Online-access only p 183-188, 2011

13. Billy GG, Miller SJ: Neurologic Thoracic Outlet Syndrome: A Case Report and Clinical Review. International Journal of Athletic Therapy & Training 21:14-20, 2016

14. Bishop MD, Mintken PE, Bialosky JE, Clel, JA: Patient expectations of benefit from interventions for neck pain and resulting influence on outcomes. J Orthop Sports Phys Ther 43:457-465, 2013

15. Blum CL: Chiropractic and pilates therapy for the treatment of adult scoliosis. J Manipulative Physiol Ther 25:E3, 2002

16. Bordoni B, Marelli F, Morabito B, Sacconi B: Osteopathic treatment in a patient with left-ventricular assist device with left brachialgia: a case report. Int Med Case Rep J 10:19-23, 2017

17. Br, t JR: C5-C6 and C6-C7 disc herniation with stenosis causing nerve root. J Acad Chiropr Orthoped (JACO) 8:6-11, 2011

18. Brouillette DL, Gurske DT: Chiropractic treatment of cervical radiculopathy caused by a herniated cervical disc [case report]. J Manipulative Physiol Ther 17:119-123, 1994

19. Buchmann J, Wende K, Kundt G, Haessler F: Manual treatment effects to the upper cervical apophysial joints before, during, and after endotracheal anesthesia: a placebo-controlled comparison. American Journal of Physical Medicine & Rehabilitation 84:251-257, 2005

20. Burcon M, Pero J: Resolution of Glossopharyngeal neuralgia & spastic dystonia following chiropractic care to reduce upper cervical vertebral subluxation: A case study [case report]. J Upper Cervical Chiropr Res 2014:Online-access only p7-13, 2014

21. Burcon MT: Resolution of trigeminal neuralgia following chiropractic care to reduce cervical spine vertebral subluxations: a case study. Journal of Vertebral Subluxation Research (JVSR):1-7, 2009

22. Chen Q, Feng JF, Tang X, Li YL, Chen L, Chen G: Cervical epidural hematoma after spinal manipulation therapy: a case report. BMC Musculoskelet Disord 20:461, 2019

23. Chen YY, Chai HM, Wang CL, Shau YW, Wang SF: Asymmetric thickness of oblique capitis inferior and cervical kinesthesia in patients with unilateral cervicogenic headache. J Manipulative Physiol Ther 41:680-690, 2018

24. Cheshire A, Polley M, Peters D, Ridge D: Is it feasible and effective to provide osteopathy and acupuncture for patients with musculoskeletal problems in a GP setting? A service evaluation. BMC Fam Pract 12:49, 2011

25. Cicconi M, Mangiulli T, Bolino G: Onset of complications following cervical manipulation due to malpractice in osteopathic treatment: a case report. Med Sci Law 54:230-233, 2014

26. Comley L: Chiropractic management of greater occipital neuralgia. Clinical Chiropractic 6:120-128, 2003

27. Coulis CM, Lisi AJ: Chiropractic management of postoperative spine pain: a report of 3 cases. Journal of Chiropractic Medicine 12:168-175, 2013

28. Cox JM: Failed back surgical syndrome - L1-2 and L5-S1 disc herniations following L4- S1 spinal fusion: A case report [case report]. J Acad Chiropr Orthoped (JACO) 6:Online-access only p, 2009

29. Crane P, Ladden J, Monica D: Treatment of axillary web syndrome using instrument assisted soft tissue mobilization and thoracic manipulation for associated thoracic rotation dysfunction. Physiother Theory Pract 34:74-78, 2018

30. Cremata E, Collins S, Clauson W, Solinger AB, Roberts ES: Manipulation under anesthesia: a report of four cases. Journal of Manipulative & Physiological Therapeutics 28:526-533, 2005

31. Cuka C, McDevitt AW, Porter-Hoke A, Karas S: Spinal manipulation after multiple fusions in an adult with scoliosis: a case report. Journal of Manual & Manipulative Therapy (Maney Publishing) 27:115-124, 2019

32. Dabbert O, Freeman DG, Weis AJ: Spinal meningeal hematoma, warfarin therapy, and chiropractic adjustment. Jama 214:2058, 1970

33. Demirel A, Oz M, Ulger O: The effect of minimal invasive techniques and physiotherapy on pain and disability in elderly: A retrospective study. Journal of Back & Musculoskeletal Rehabilitation 32:63-70, 2019

34. Dennis AK, Oakley PA, Weiner MT, VanVranken TA, Shapiro DA, Harrison DE: Alleviation of neck pain by the non-surgical rehabilitation of a pathologic cervical kyphosis to a normal lordosis: a CBP(®) case report. J Phys Ther Sci 30:654-657, 2018

35. Destee A, Lesoin F, Di Paola F, Warot P: Intradural herniated cervical disc associated with chiropractic spinal manipulation. J Neurol Neurosurg Psychiatry 52:1113, 1989

36. Devèze A, Alimi Y, Tardivet L, Lavieille JP, Magnan J: Surgical management of lesions of the internal carotid artery using a modified Fisch type A infratemporal approach. Otol Neurotol 28:94-99, 2007

37. Dion L, Rodgers N, Cutshall SM, Cordes ME, Bauer B, Cassivi SD, et al: Effect of massage on pain management for thoracic surgery patients. Int J Ther Massage Bodywork 4:2-6, 2011

38. Domenicucci M, Ramieri A, Salvati M, Brogna C, Raco A: Cervicothoracic epidural hematoma after chiropractic spinal manipulation therapy. Case report and review of the literature. J Neurosurg Spine 7:571-574, 2007

39. Dutta G, Jagetia A, Srivastava AK, Singh D, Singh H, Saran RK: "Crick" in Neck Followed by Massage Led to Stroke: Uncommon Case of Vertebral Artery Dissection. World Neurosurg 115:41-43, 2018

40. Eisenberg EbE, Vulfsons S, Waldman M: Persistent Back Pain after Multiple Operations: Case Presentation From Israel With Commentaries From Austria and The Netherlands. Journal of Pain & Palliative Care Pharmacotherapy 25:64-67, 2011

41. Ekman P, Moller H, Hedlund R: The long-term effect of posterolateral fusion in adult isthmic spondylolisthesis: a randomized controlled study. The Spine Journal 2005;5(1):36-44, 2005

42. Epstein NE, Forte Esq CL: Medicolegal corner: Quadriplegia following chiropractic manipulation. Surg Neurol Int 4:S327-329, 2013

43. Eriksen K: Management of cervical disc herniation with upper cervical chiropractic care. Journal of Manipulative & Physiological Therapeutics 21:51-56, 1998

44. Eugene C: Chiropractic management of a 30-year-old patient with Parsonage-Turner syndrome. Journal of Chiropractic Medicine 10:301-305, 2011

45. Fattahi A, Taheri M: Spontaneous resolved cervical spine epidural hematoma: A case report. Surg Neurol Int 8:183, 2017

46. Fedorchuk C, Lightstone D: Reduction in cervical anterolisthesis & pain in a 52-year-old female using Chiropractic BioPhysics® Technique: A case study and selective review of literature [case report]. Ann Vert Sublux Res 2016:Online-access only p 118-124, 2016

47. Fedorchuk C, Lightstone DF, Andino H: Failed neck surgery: Improvement in neck pain, migraines, energy levels, and performance of activities of daily living following subluxation correction using Chiropractic BioPhysics® Technique: A case study [case report]. Ann Vert Sublux Res 2017:Online-access only p 93-100, 2017

48. Fırat T, Sağlam M, Vardar Yağlı N, Tunç Y, Çalık Kütükçü E, Delioğlu K, et al: Acute effects of manual therapy on respiratory parameters in thoracic outlet syndrome. Turk Gogus Kalp Damar Cerrahisi Derg 27:101-106, 2019

49. Forte ML, Maiers M: Differences in Function and Comorbidities Between Older Adult Users and Nonusers of Chiropractic and Osteopathic Manipulation: A Cross-sectional Analysis of the 2012 National Health Interview Survey. J Manipulative Physiol Ther 42:450-460, 2019

50. Fortner MO, Oakley PA, Harrison DE: Cervical extension traction as part of a multimodal rehabilitation program relieves whiplash-associated disorders in a patient having failed previous chiropractic treatment: a CBP(®) case report. J Phys Ther Sci 30:266-270, 2018

51. Fortner MO, Oakley PA, Harrison DE: Chiropractic Biophysics management of straight back syndrome and exertional dyspnea: A case report with follow-up. J Contemp Chiropr 2:Online-access only p 115-122, 2019

52. Fortner MO, Oakley PA, Harrison DE: Non-surgical improvement of cervical lordosis is possible in advanced spinal osteoarthritis: a CBP(®) case report. J Phys Ther Sci 30:108-112, 2018

53. Fortner MO, Oakley PA, Harrison DE: Treating 'slouchy' (hyperkyphosis) posture with chiropractic biophysics(®): a case report utilizing a multimodal mirror image(®) rehabilitation program. J Phys Ther Sci 29:1475-1480, 2017

54. Friedman R, Friedman A: Resolution of Vasovagal Syncope (VVS) following upper cervical chiropractic care: A case study and review of the literature [case report; review]. J Upper Cervical Chiropr Res 2018:Online-access only p 1-12, 2018

55. Garg P: Home care network traction for a patient with neck pain and cervical radiculopathy symptoms: A case report. J Chiropr Med 18:127-130, 2019

56. Garrigós-Pedrón M, La Touche R, Navarro-Desentre P, Gracia-Naya M, Segura-Ortí E: Effects of a Physical Therapy Protocol in Patients with Chronic Migraine and Temporomandibular Disorders: A Randomized, Single-Blinded, Clinical Trial. Journal of Oral & Facial Pain & Headache 32:137-150, 2018

57. Gundersen B: Cervical disc disease with radiculopathy recommended surgery, responds to conservative measures [case report]. J Acad Chiropr Orthoped (JACO) 9:Online-access only p 3-5, 2012

58. Hardas GM, Murrell GAC: Prospective, Randomized, Double-Blind, Placebo-Controlled Clinical Trial Assessing the Effects of Applying a Force to C5 by a Mechanically Assisted Instrument on Referred Pain to the Shoulder. Spine (03622436) 43:461-466, 2018

59. Hauswirth J, Ernst MJ, Preusser ML, Meichtry A, Kool J, Crawford RJ: Immediate effects of cervical unilateral anterior-posterior mobilisation on shoulder pain and impairment in post-operative arthroscopy patients. Journal of Back & Musculoskeletal Rehabilitation 30:615-623, 2017

60. Heiner JD: Cervical epidural hematoma after chiropractic spinal manipulation. Am J Emerg Med 27:1023.e1021-1022, 2009

61. Hoiriis KT: Case report: management of post-surgical chronic low back pain with upper cervical adjustment. CRJ: Chiropractic Research Journal 1:37-42, 1989

62. Houle S, Descarreaux M: Conservative care of temporomandibular joint disorder in a 35-year-old patient with spinal muscular atrophy type III: a case study. Journal of Chiropractic Medicine 8:187-192, 2009

63. Hsieh JH, Wu CT, Lee ST: Cervical intradural disc herniation after spinal manipulation therapy in a patient with ossification of posterior longitudinal ligament: a case report and review of the literature. Spine (Phila Pa 1976) 35:E149-151, 2010

64. Huang M, Barber SM, Moisi M, Powell S, Rivera A, Zwillman M, et al: Cervical Epidural Hematoma after Chiropractic Spinal Manipulation Therapy in a Patient with an Undiagnosed Cervical Spinal Arteriovenous Malformation. Cureus 7:e307, 2015

65. Jaeger B: Are "cervicogenic" headaches due to myofascial pain and cervical spine dysfunction? Cephalalgia 9:157-164, 1989

66. Jalal Y, Ahmad A, Rahman AU, Daud M: Effectiveness of muscle energy technique on cervical range of motion and pain. J Pak Med Assoc 68:811-813, 2018

67. Jentzen JM, Amatuzio J, Peterson GF: Complications of cervical manipulation: a case report of fatal brainstem infarct with review of the mechanisms and predisposing factors. J Forensic Sci 32:1089-1094, 1987

68. Kadanka Z, Bednarik J, Vohanka S, Vlach O: Spondylotic cervical myelopathy: conservative versus surgical treatment. Scripta Medica 1997;70(7):317-327, 1997

69. Kaufman MR, Elkwood AI, Rose MI, Patel T, Ashinoff R, Saad A, et al: Reinnervation of the paralyzed diaphragm: application of nerve surgery techniques following unilateral phrenic nerve injury. Chest 140:191-197, 2011

70. Kennedy L, Gonzales E, Corbin L: The Effect of Curanderismo on Chronic Non-malignant Pain: A Case Report. Explore (NY) 12:263-267, 2016

71. Kennell KA, Daghfal MM, Patel SG, DeSanto JR, Waterman GS, Bertino RE, et al: Cervical artery dissection related to chiropractic manipulation: One institution's experience: This study suggests that patients considering chiropractic cervical spine manipulation should be advised of the risks of potential arterial dissection and stroke. Journal of Family Practice 66:556-562, 2017

72. Kewalramani LS, Kewalramani DL, Krebs M, Saleem A: Myelopathy following cervical spine manipulation. Am J Phys Med 61:165-175, 1982

73. Kondo E: Nonextraction and nonsurgical treatment of an adult with skeletal Class II open bite with severe retrognathic mandible and temporomandibular disorders. World J Orthod 8:261-276, 2007

74. Kruse RA, Okamoto CS: Chiropractic Management of Cervicalgia in a Patient with Diffuse Idiopathic Skeletal Hyperostosis Utilizing Cox Manual Cervical Distraction: A Case Report. Journal of the Academy of Chiropractic Orthopedists 14:21-31, 2017

75. Lee S, Hanks KB, Schwartz J: Pianist's rehabilitation: three cases. Medical Problems of Performing Artists 20:35-39, 2005

76. Lee TH, Chen CF, Lee TC, Lee HL, Lu CH: Acute thoracic epidural hematoma following spinal manipulative therapy: case report and review of the literature. Clin Neurol Neurosurg 113:575-577, 2011

77. Lee T-H, Chiu J-W, Chan R-C: Cervical Cord Injury After Massage. American Journal of Physical Medicine & Rehabilitation 90:856-859, 2011

78. Lewit K, Olsanska S: Clinical importance of active scars: abnormal scars as a cause of myofascial pain. J Manipulative Physiol Ther 27:399-402, 2004

79. Liao CC, Chen LR: Anterior and posterior fixation of a cervical fracture induced by chiropractic spinal manipulation in ankylosing spondylitis: a case report. J Trauma 63:E90-94, 2007

80. Licht PB, Christensen HW, Høilund-Carlsen PF: Is cervical spinal manipulation dangerous? Journal of Manipulative & Physiological Therapeutics 26:48-52, 2003

81. Lidder S, Lang KJ, Masterson S, Blagg S: Acute spinal epidural haematoma causing cord compression after chiropractic neck manipulation: an under-recognised serious hazard? J R Army Med Corps 156:255-257, 2010

82. Liu HP, Chen CL, Chen NF, Liao CY, Ou CY: Ligamentum flavum hematoma due to stretching exercise. Am J Emerg Med 34:2058.e2053-2058.e2056, 2016

83. Liu X, Liu S: Evaluation of therapeutic effect of maneuver-dominated method in 30 cases of cervical spondylotic myelopathy. J Tradit Chin Med 20:282-286, 2000

84. Mann DJ, Mattox R: Chiropractic Management of a Patient With Chronic Pain in a Federally Qualified Health Center: A Case Report. Journal of Chiropractic Medicine 17:117-120, 2018

85. Mathews RS, Miller MR, West DT: Effective management of spinal pain in one hundred seventy-seven patients evaluated for manipulation under anesthesia. J Manipulative Physiol Ther 22:299-308, 1999

86. McAninch S, Martin J: Symptomatic Arachnoid Cyst After Cervical Manipulation. J Emerg Med 55:845-847, 2018

87. Mikkelsen R, Dalby RB, Hjort N, Simonsen CZ, Karabegovic S: Endovascular Treatment of Basilar Artery Thrombosis Secondary to Bilateral Vertebral Artery Dissection with Symptom Onset Following Cervical Spine Manipulation Therapy. Am J Case Rep 16:868-871, 2015

88. Min SH, Chang SH, Jeon SK, Yoon SZ, Park JY, Shin HW: Posterior auricular pain caused by the trigger points in the sternocleidomastoid muscle aggravated by psychological factors -A case report. Korean J Anesthesiol 59:S229-232, 2010

89. Mitchinson AR, Kim HM, Rosenberg JM, Geisser M, Kirsh M, Cikrit D, et al: Acute postoperative pain management using massage as an adjuvant therapy: a randomized trial. Arch Surg 142:1158-1167; discussion 1167, 2007

90. Moon K, Albuquerque FC, Cole T, Gross BA, McDougall CG: Stroke prevention by endovascular treatment of carotid and vertebral artery dissections. Journal of NeuroInterventional Surgery 9:1-7, 2017

91. Morningstar MW, Joy T: Scoliosis treatment using spinal manipulation and the Pettibon Weighting System: a summary of 3 atypical presentations. Chiropr Osteopat 14:1, 2006

92. Neetu R, Ch, ra MS, Rashmi M: Cervical spinal epidural hematoma with acute Brown-Séquard presentation. Neurol India 54:107-108, 2006

93. Neff SM, Schielke AL: Chiropractic Management of a Patient with Chronic Post-surgical Neck Pain: A Case Report. Journal of the Academy of Chiropractic Orthopedists 13:2-7, 2016

94. Oakley PA, Harrison DE: Alleviation of pain and disability in a post-surgical C4-C7 total fusion patient after reducing a lateral head translation (side shift) posture: a CBP(®) case report with a 14 year follow-up. J Phys Ther Sci 30:952-957, 2018

95. Oppenheim JS, Spitzer DE, Segal DH: Nonvascular complications following spinal manipulation. Spine J 5:660-666; discussion 666-667, 2005

96. Pederick FO: Spinal stenosis: case report with a review of the literature. Chiropractic Journal of Australia 31:82-91, 2001

97. Pringle RK, Richardson DL, Shiel RS: Case report: myofascial pain syndrome: a double crush-like appearance. Journal of Chiropractic Medicine 2:66-74, 2003

98. Ranganath PNU, Dowle P, Ch, rasekhar P: Effectiveness of MWM, neurodynamics and conventional therapy versus neurodynamics and conventional therapy in unilateral cervical radiculopathy: a randomized control trial. Indian Journal of Physiotherapy and Occupational Therapy 2018 Jul-Sep;12(3):101-106, 2018

99. Salame K, Grundshtein A, Regev G, Khashan M, Lador R, Lidar Z: Acute Presentation of Cervical Myelopathy Following Manipulation Therapy. Isr Med Assoc J 21:542-545, 2019

100. Schneider M, Santolin S, Farrell P: False negative magnetic resonance imaging results: a report of 2 cases. Journal of Manipulative & Physiological Therapeutics 28:278-284, 2005

101. Sedat J, Chau Y, Mahagne MH, Bourg V, Lonjon M, Paquis P: Dissection of the posteroinferior cerebellar artery: clinical characteristics and long-term follow-up in five cases. Cerebrovasc Dis 24:183-190, 2007

102. Simnad VI: Acute onset of painful ophthalmoplegia following chiropractic manipulation of the neck. Initial sign of intracranial aneurysm. West J Med 166:207-210, 1997

103. Sorrell MR, Flanagan W: Treatment of chronic resistant myofascial pain using a multidisciplinary protocol [The Myofascial Pain Program]. Journal of Musculoskeletal Pain 11:5-9, 2003

104. Sorrell MR, Flanagan W, McCall JL: Symptom duration affects the outcome of multidisciplinary treatment of myofascial pain: the method of assessment influences the understanding of the results. Journal of Musculoskeletal Pain 11:11-16, 2003

105. Stuart PJ, Bernstein T: A case of subdural hematoma and temporal bone fracture as complications of chiropractic manipulation. J Emerg Med 7:615-617, 1989

106. Talluri SK, Talluri J, Besur S, Kakarala R, Klair N: Catastrophic complication of chiropractic manipulation: a report of quadriparesis. American Journal of Medicine 122:e3-4, 2009

107. Teixeira MJ, Yeng LT, Garcia OG, Fonoff ET, Paiva WS, Araujo JO: Failed back surgery pain syndrome: therapeutic approach descriptive study in 56 patients. Rev Assoc Med Bras (1992) 57:282-287, 2011

108. Tseng SH, Chen Y, Lin SM, Wang CH: Cervical epidural hematoma after spinal manipulation therapy: case report. J Trauma 52:585-586, 2002

109. Tseng SH, Lin SM, Chen Y, Wang CH: Ruptured cervical disc after spinal manipulation therapy: report of two cases. Spine (Phila Pa 1976) 27:E80-82, 2002

110. Tsou A, Juan Y-H, Chen T-Y, Lin S-K: Thrombolysis for atlantoaxial dislocation mimicking acute ischemic stroke. American Journal of Emergency Medicine 37:1216.e1213-1216.e1215, 2019

111. Veena Kirthika S, Padmanabhan K, Sudhakar S, Vijaya Kumar M: Is Mulligan's sustained natural apophyseal glides (SNAGS) or muscle energy technique is effective in the non-surgical management of cervicogenic headache? A two-group pretest-posttest randomized controlled trial. Asian Journal of Pharmaceutical and Clinical Research 2018 Sep;11(9):230-233, 2018

112. Vibert D, Rohr-Le Floch J, Gauthier G: Vertigo as manifestation of vertebral artery dissection after chiropractic neck manipulations. ORL J Otorhinolaryngol Relat Spec 55:140-142, 1993

113. Waldrop MA: Diagnosis and treatment of cervical radiculopathy using a clinical prediction rule and a multimodal intervention approach: a case series. J Orthop Sports Phys Ther 36:152-159, 2006

114. Wang CC, Kuo JR, Chio CC, Tsai TC: Acute paraplegia following chiropractic therapy. J Clin Neurosci 13:578-581, 2006

115. Weiner MT, Oakley PA, Dennis AK, Shapiro DA, Harrison DE: Increasing the cervical and lumbar lordosis is possible despite overt osteoarthritis and spinal stenosis using extension traction to relieve low back and leg pain in a 66-year-old surgical candidate: a CBP(®) case report. J Phys Ther Sci 30:1364-1369, 2018

116. Whedon JM, Quebada PB, Roberts DW, Radwan TA: Spinal epidural hematoma after spinal manipulative therapy in a patient undergoing anticoagulant therapy: a case report. J Manipulative Physiol Ther 29:582-585, 2006

117. Wilding LJ, Howlett DC, Anderson HJ, Sangle PD, Violaris N, Evans GH: Extracranial internal carotid artery aneurysm presenting as symptomatic hypoglossal and glossopharyngeal nerve paralysis. J Laryngol Otol 118:150-152, 2004

118. Wilson D, Steel T, Sutton I: Surgical treatment of cervical disc protrusion causing intracranial hypotension following chiropractic manipulation. J Clin Neurosci 22:1523-1525, 2015

119. Wise R: Seventh cervical rib associated with subclavian artery occlusion and multiple infarcts: case report. Journal of Neuroscience Nursing 40:169-172, 2008

120. Wong LC: Rehabilitation of a patient with a rare multi-level isthmic spondylolisthesis: a case report. J Can Chiropr Assoc 48:142-151, 2004

121. Zeng YJ: Persistent Idiopathic Facial Pain Originating from Cervical Abnormalities. World Neurosurg 133:248-252, 2020

122. Zupruk GM, Mehta Z: Brown-Séquard syndrome associated with posttraumatic cervical epidural hematoma: case report and review of the literature. Neurosurgery 25:278-280, 1989

**No manual therapy (17)**

1. Aspegren DD, Burt AL: A study of postspinal surgery cases in chiropractic offices. J Manipulative Physiol Ther 17:88-92, 1994

2. Cates JR, Soriano MM: Cervical spondylotic myelopathy [case report]. J Manipulative Physiol Ther 18:471-475, 1995

3. Chen HC, Hsu PW, Lin CY, Tzaan WC: Symptomatic hematoma of cervical ligamentum flavum: case report. Spine (Phila Pa 1976) 30:E489-491, 2005

4. Chen WF, Kang CJ, Lee SC, Tsao CK: Quadriplegia secondary to cervical spondylotic myelopathy-a rare complication of head and neck surgery. Head Neck 35:E49-51, 2013

5. De Gelb D, Lenke L, Pond J: Dural tear associated with a flexion distraction subluxation to the cervical spine without neurologic injury. Acta Orthop Belg 64:224-228, 1998

6. Engquist M, Löfgren H, Öberg B, Holtz A, Peolsson A, Söderlund A, et al: Surgery versus nonsurgical treatment of cervical radiculopathy: a prospective, randomized study comparing surgery plus physiotherapy with physiotherapy alone with a 2-year follow-up. Spine (Phila Pa 1976) 38:1715-1722

7. Franklin A: Post-surgical cervical myelopathy. Eur J Chiropr 46:41-48, 1998

8. Golub D, Hu L, Dogra S, Torres J, Shapiro M: Spontaneous bilateral internal carotid and vertebral artery dissections with dominant-hemisphere circulation maintained by external carotid artery-ophthalmic artery anastomoses. Neurosurg Focus 46:E6, 2019

9. Graff-Radford SB, Jaeger B, Reeves JL: Myofascial pain may present clinically as occipital neuralgia. Neurosurgery 19:610-613, 1986

10. King K, Pollard H, Gordon B: Cervical Spondylotic Myelopathy: A Review and Case Report. Chiropractic Journal of Australia 41:99-105, 2011

11. Lapeer GL: Postsurgical myofascial pain resolved with dry-needling. Treatment protocl and case report. Cranio 7:243-244, 1989

12. McGregor AH, Henley A, Morris TP, Dore CJ: An evaluation of a postoperative rehabilitation program after spinal surgery and its impact on outcome [with consumer summary]. Spine 2012 Apr 1;37(7):E417-E422, 2012

13. Peolsson A, Kjellman G: Neck muscle endurance in nonspecific patients with neck pain and in patients after anterior cervical decompression and fusion. J Manipulative Physiol Ther 30:343-350, 2007

14. Peolsson A, Lofgren H, Dedering A, Oberg B, Zsigmond P, Hedevik H, et al: Postoperative structured rehabilitation in patients undergoing surgery for cervical radiculopathy: a 2-year follow-up of a randomized controlled trial. Journal of Neurosurgery Spine 2019 Jul;31(1):60-69, 2019

15. Peolsson AL, Peolsson MN, Jull GA, O'Leary SP: Cervical muscle activity during loaded arm lifts in patients 10 years postsurgery for cervical disc disease. J Manipulative Physiol Ther 36:292-299, 2013

16. Wibault J, Oberg B, Dedering A, Lofgren H, Zsigmond P, Peolsson A: Structured postoperative physiotherapy in patients with cervical radiculopathy: 6-month outcomes of a randomized clinical trial. Journal of Neurosurgery Spine 2018 Jan;28(1):1-9, 2018

17. Wibault J, Öberg B, Dedering A, Löfgren H, Zsigmond P, Persson L, et al: Neck-related physical function, self-efficacy, and coping strategies in patients with cervical radiculopathy: A randomized clinical trial of postoperative physiotherapy. J Manipulative Physiol Ther 40:330-339, 2017

18. Svensson J, Hermansen A, Wibault J, Löfgren H, Dedering Å, Öberg B, Zsigmond P, Peolsson A. Neck-Related Headache in Patients With Cervical Disc Disease After Surgery and Physiotherapy: A 1-Year Follow-up of a Prospective Randomized Study. Spine (Phila Pa 1976). 2020 Jul 15;45(14):952-959.

**Foreign language (7)**

1. Altomare GRS, Pereira JS: Aplication of the general osteopatic treatment post cervical discectomy: a case study [sic]. Revista Terapia Manual 7:216-220, 2009

2. Bien JY, Morel J, Demasles S, Abboud K, Molliex S: [Postoperative dissection of the vertebral artery in two steps]. Ann Fr Anesth Reanim 33:696-699, 2014

3. Collado Cruz A, Torres i Mata X, Arias i Gassol A, Cerdà Gabaroi D, Vilarrasa R, Valdés Miyar M, et al: [Efficiency of multidisciplinary treatment of chronic pain with locomotor disability]. Med Clin (Barc) 117:401-405, 2001

4. Ding HT, Tang XZ: [Study on the clinical effect of the massage method of micro-regulating with vertical cross pressing lying on one side in treating cervicogenic headache]. Zhongguo Gu Shang 28:722-726, 2015

5. Gusarova SA, Kuznetsov OF, Gorbunov FE, Maslovskaia SG: [The methodological aspects of using cryomassage on patients operated on for discogenic neuropathies]. Vopr Kurortol Fizioter Lech Fiz Kult:20-22, 2000

6. Leonelli C, Zucchini E, Messora A, Sartini S, Fontana L, Parazza S: Neurodynamic technique benefits in patients with chronic cervical radiculopathy: a pilot study. Scienza Riabilitativa 15:19-28, 2013

7. Pennella D, Raposio E, Mourad F, Cataldi F, Maselli F: LA TERAPIA MANUALE MIGLIORA GLI OUTCOME DI UN PAZIENTE CON CEFALEA CERVICOGENICA CRONICA RESISTENTE A TRATTAMENTO CHIRURGICO. UN CASO CLINICO. Scienza Riabilitativa 20:21-29, 2018

**Wrong publication type (6)**

1. Downie PA: The rehabilitation for patients following head and neck surgery. J Laryngol Otol 89:1281-1284, 1975

2. Garvey C: Thoracic manual therapy for cervical pain in a patient with a multilevel cervical fusion: a case report. Journal of Orthopaedic & Sports Physical Therapy 39:A81-82, 2009

3. Imamura ST: Efficacy of myofascial therapy in failed back surgery. Acupuncture & Electro-Therapeutics Research 31:163-164, 2006

4. Prasanna A: Myofascial pain as postoperative complication. J Pain Symptom Manage 8:450-451, 1993

5. Stanworth PA: Acute spinal epidural haematoma causing cord compression after chiropractic neck manipulation: an under-recognised serious hazard? J R Army Med Corps 157:197;-author reply 197, 2011

6. Winterstein JF, Plaugher G, Troyanovich SJ, Alcantara J, Thornton R, Coleman RR: Chiropractic care of a patient with vertebral subluxations and unsuccessful surgery of the cervical spine...case study presented by Drs Joel Alcantara, Gregory Plaugher, Richard Thornton, and Chris Salem. Journal of Manipulative & Physiological Therapeutics 25:283-284, 2002

**Wrong study design (4)**

1. Béjot Y, Aboa-Eboulé C, Debette S, Pezzini A, ro, Tatlisumak T, et al: Characteristics and outcomes of patients with multiple cervical artery dissection. Stroke (00392499) 45:37-41, 2014

2. Elkayam O, Ben Itzhak S, Avrahami E, Meidan Y, Doron N, Eldar I, et al: Multidisciplinary approach to chronic back pain: prognostic elements of the outcome. Clin Exp Rheumatol 14:281-288, 1996

3. Jonely H, Scalzitti DA: In a 36-Year-Old Woman With Neck Pain, Will Manipulation and Mobilization Be Beneficial for Reducing Her Reports of Neck Pain? Physical Therapy 94:179-184, 2014

4. Moskovich R: Neck pain in the elderly: common causes and management. Geriatrics 43:65-82, 1988

**< 18 years of age (3)**

1. Brown J, Chung J, O'Connell K: Complications following brain surgery improved after upper cervical chiropractic care: A case study [case report]. J Upper Cervical Chiropr Res 2013:Online-access only p 43-48, 2013

2. Burdi M, Bono CM, Kauffman CP, Hoyt D, Garfin SR: Delayed diagnosis of a flexion-distraction (seat belt) injury in a patient with multiple abdominal injuries: a case report. Am J Orthop (Belle Mead NJ) 37:44-46, 2008

3. Remvig L, Jensen KE, Lind M, Jansen TU, Andersen GR, Christensen SB: Persistent atlanto-axial rotary subluxation with zygapophyseal joint surface deformation, successfully treated with traction, immobilisation and manual medicine. International Musculoskeletal Medicine 30:55-60, 2008

**No outcomes (2)**

1. Christensen KD, Buswell K: Chiropractic outcomes managing radiculopathy in a hospital setting: a retrospective review of 162 patients. Journal of Chiropractic Medicine 7:115-125, 2008

2. Dellamonte NA: The alteration of spinal biomechanics after bilateral posterior laminectomy and fusion with instrumentation. Chiropractic Technique 9:62-66, 1997

**Wrong outcome (1)**

1. Herman PM, Kommareddi M, Sorbero ME, Rutter CM, Hays RD, Hilton LG, et al: Characteristics of Chiropractic Patients Being Treated for Chronic Low Back and Neck Pain. Journal of Manipulative & Physiological Therapeutics 41:445-455, 2018
